# The Disappearance of Respiratory Viruses in Children during the COVID-19 Pandemic

**DOI:** 10.3390/ijerph18189550

**Published:** 2021-09-10

**Authors:** Anna Chiara Vittucci, Livia Piccioni, Luana Coltella, Claudia Ciarlitto, Livia Antilici, Elena Bozzola, Fabio Midulla, Paolo Palma, Carlo Federico Perno, Alberto Villani

**Affiliations:** 1 General Pediatrics Unit, Pediatric Emergency and General Pediatrics Department, Bambino Gesù Children’s Hospital, IRCCS, 00165 Rome, Italy; claudia.ciarlitto@opbg.net (C.C.); livia.antilici@opbg.net (L.A.); elena.bozzola@opbg.net (E.B.); alberto.villani@opbg.net (A.V.); 2 Unit of Microbiology and Diagnostic Immunology, Department of Diagnostic and Laboratory Medicine, Bambino Gesù Children’s Hospital, IRCCS, 00165 Rome, Italy; livia.piccioni@opbg.net (L.P.); luana.coltella@opbg.net (L.C.); carlofederico.perno@opbg.net (C.F.P.); 3 Department of Maternal Infantile and Urological Sciences, Sapienza University of Rome, 00161 Rome, Italy; midulla@uniroma1.it; 4 Clinical Immunology and Vaccinology Unit, Academic Department of Pediatrics, Bambino Gesù Children’s Hospital, IRCCS, 00165 Rome, Italy; paolo.palma@opbg.net

**Keywords:** social distancing, COVID-19, viral infections, pandemic, respiratory viruses, childhood

## Abstract

Background: Social distancing measures are used to reduce the spreading of COVID-19. The aim of this study was to assess the impact of local restrictions on the transmission of respiratory virus infections. Methods: we retrospectively analyzed the nasopharyngeal samples of all patients (0–18 years old) admitted with respiratory symptoms in a large Italian tertiary hospital during the last three seasons from 2018 to 2021. Results: A strong reduction in all viral respiratory infections was observed in the last season (2020–2021) compared to the two previous seasons (−79.69% and −80.66%, respectively). In particular, we found that during the epidemic period 2018–2019 and 2019–2020, the total number of Respiratory Syncytial Virus (RSV) cases was, respectively 726 and 689, while in the last season a total of five cases was detected. In the first months of 2018–2019 and 2019–2020, the total flu infections were 240 and 354, respectively, while in the last season we did not detect any influenza virus. As other viruses, the presence of Rhinovirus declined, but to a lesser extent: a total of 488 cases were assessed compared to the 1030 and 1165 cases of the two previous respective epidemic seasons. Conclusions: Public health interventions and distancing (including continuous use of face masks) settled to counter the pandemic spread of COVID-19 had a macroscopic impact on all respiratory virus transmission and related diseases, with a partial exception of Rhinovirus. The absence of viruses’ circulation could result in a lack of immunity and increased susceptibility to serious infections in the next seasons.

## 1. Introduction

Globally, acute respiratory infections (ARIs) remain one of the leading causes of morbidity and mortality in children younger than 5 years [1]. They occur mainly in children and infants, who can experience up to five to six episodes in any given year [1]. For this reason, ARIs represent a persistent public health problem. Although the majority of ARIs remain confined to the upper respiratory tract, they can cause severe manifestations when they affect the lower respiratory tract [2]. Viruses represent approximately 90% of all respiratory tract infections with the subsequent 10% of infections caused by bacteria [3].

Respiratory syncytial virus (RSV) is one of the most common viral pathogens identified in children with ARI, but other frequent viruses are Rhinoviruses (HRV), Influenza viruses (flu), Enteroviruses (EV), Adenoviruses (ADV), Parainfluenza viruses (PIV), Coronaviruses (CoV), Human Metapneumovirus (MPV), and Bocavirus (BoV).

In 2020, the novel coronavirus (SARS-CoV-2) pandemic spread worldwide [4]; the World Health Organization and national governments have recommended a series of measures to prevent the infection. Italy was the first country affected by SARS-CoV-2 in Europe, in February 2020. The Italian government has imposed a broad range of public health measures to contain the pandemic, upgraded from strict social distancing and school closures on 4 March 2020 and culminating with the national lockdown on 11 March 2020, terminated on 4 May 2020. After the nationwide lockdown, facial masks, social distancing, remote work, stay at home orders, and travel restrictions were strongly recommended. 

The preventive measures useful in containing COVID-19 spreading were not specific to SARS-CoV-2 and seemed to be effective in preventing transmission of other respiratory viruses. Matera et al. demonstrated that during the lockdown period, Emergency Room admissions decreased by 81% compared to 2019, and this reduction was significantly higher for air communicable diseases [5]. Raucci et al. also demonstrated a strong reduction in visits in Emergency Department for Respiratory Diseases [6].

The aim of this study was to compare the frequency of respiratory virus infections in the last year (characterized by COVID-19 pandemic) to the previous two COVID-free seasons.

## 2. Methods

This retrospective study involved all patients (0–18 years old) admitted with respiratory symptoms at Bambino Gesù Children’s Hospital in Rome during the last three winter seasons (September to February) from 2018 to 2021. Samples collected included nasopharingeal swabs or aspirate, and were processed, immediately or after storage at −80 °C. The identification of respiratory viruses was accomplished by the multiplex RT-PCR “AllplexTM Respiratory Panel Assays” on All-in-One Platform (Seegene, Korea). Nucleic acids were extracted using the STARMag Universal Cartridge Kit (Seegene, Korea) on the automated Nimbus IV platform that can process 30 samples per run. Two hundred microliters of sample were extracted, and nucleic acid was eluted with 100 μL of elution buffer. Real time PCR was performed on CFX96 (Bio Rad Laboratories), for each reaction 8 μL of the extracted DNA/RNA in a final volume of 25 μL were used. The panel is composed of 3 multiplex PCR for the detection of 16 different viruses (Influenza A and B virus, Respiratory syncytial virus A and B, Adenovirus, Enterovirus, Parainfluenza virus 1, 2, 3, and 4, Metapneumovirus, Bocavirus, Rhinovirus, and 3 Coronaviruses NL63/ 229E/OC43). An internal control was included in each sample to check both extraction efficiency and PCR inhibition. In every run, a negative control was used to monitor carry-over contamination and a positive control to check PCR reaction. The results were analyzed automatically using Seegene software (Seegene Viewer V2.0, Seoul, Korea). According to datasheet indications for results interpretation, samples with a Cycle threshold (Ct) ≤ 42 were considered positive; samples with no Ct or a Ct > 42 were considered negative.

The whole workflow was performed as recommended by the manufacturer.

Continuous variables were not normally distributed; thus, Kruskal–Wallis test was used to compare groups. All statistical analyses were carried out using the SPSS (Statistical Package of Social Sciences, Chicago, IL, USA) for Windows software program version 26.0. *p*-values of <0.05 were considered statistically significant.

Institutional board review was not required as no individual-level data were used.

## 3. Results

During the first season (2018–2019) we had 3029 viral infections (patients’ mean age: 1 year; range 0–18), during the second season (2019–2020) we had 3180 viral infections (patients’ mean age: 2 years; range 0–18), during the last season ravaged by COVID-19 (2020–2021) we had 615 viral infections (patients’ mean age: 2 years; range 0–18).

Table 1 shows the monthly distribution of virus detection during the three seasons.

A strong reduction was observed in the third season: −79.69% compared to 2018–2019 and −80.66% compared to 2019–2020.

**Figure 1:**
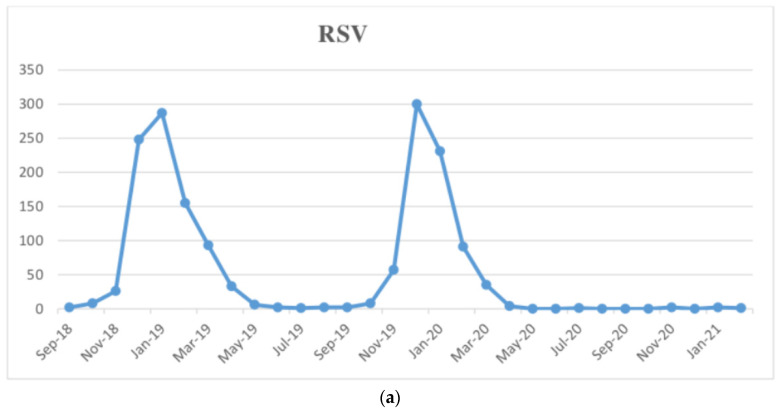

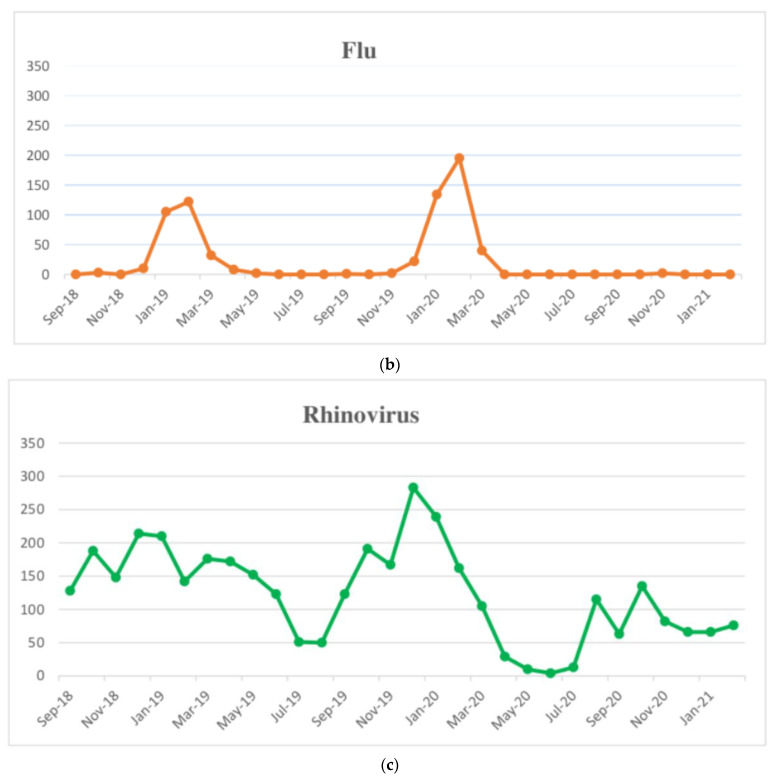
The cyclical trend of RSV (**a**), flu (**b**), and Rhinovirus (**c**) during the last 3 years.

Figure 1 shows the epidemiological curves of RSV, flu, and HRV, the three most involved respiratory viruses, during the last 3 years.

During the season 2018–2019 and 2019–2020, the total number of RSV cases was, respectively 726 and 689, while in the last season only five cases were detected. RSV detections in 2020–2021 were 99.31% and 99.27% lower compared to 2018–2019 and 2019–2020, respectively (*p* = 0.016 and *p* = 0.016).

Similarly, the total flu infections were 240 in 2018–2019 and 354 in 2019–2020, while in the last season we did not detect any case of influenza virus.

Similar to other viruses, Rhinoviruses’ (HRV) detection also declined, although to a lesser extent than flu and RSV: a total of 488 cases were assessed in 2020–2021 compared to the 1030 and 1165 cases of in 2018–2019 and 2019–2020; respectively, a 52.62% and 58.11% reduction in the number of cases was recorded. This difference remains statistically significant despite being less remarkable than that found for flu and RSV. Pairwise comparison still showed significant difference with the Bonferroni correction of the third season compared to the first two (*p* = 0.033 and *p* = 0.009, respectively).

Of interest, the incidence of HRV accounts for 79.34% of all respiratory infections in 2020–2021, a relative increase compared to 2018–2019 and 2019–2020, when HRV represented only 34.0% and 36.63%, respectively, of all the respiratory viruses detected in our hospital.

## 4. Discussion

The findings of this study suggest that the public health interventions established to contrast the pandemic spread of COVID-19 also have a critical impact on the transmission of other viral infectious diseases.

We compared the number of viral detections in children aged 0–18 years hospitalized at Bambino Gesù Children Hospital in Rome, during the last 3 years. Compared to the last two seasons, we registered a decrease of 79.69% and 80.66%, respectively, in the total number of respiratory infections. Notably, our report showed a strong reduction in RSV and flu cases. Although an important decrease in Rhinovirus cases emerged, in our patients, this latter virus becomes the main one responsible for ARI during this winter, with a total of 488 cases (79.34% of all infections in 2020–2021).

Regarding flu, it is important to underline that, during the last season (2020–2021), Italy has implemented a vaccination campaign providing free of charge influenza vaccine for children from 6 months to 6 years, to reduce the burden on healthcare systems.

RSV, similar to the other respiratory viruses, seems very sensible to the adopted mitigation strategies such as distance, hand hygiene, and massive use of facial masks [7].

It is notable that the number of RSV detected in our hospital this year was equal to five cases, with its complete disappearance as the main responsible agent for bronchiolitis.

Our results are utterly in agreement with what described in the literature. The beginning of public health interventions to contrast COVID-19 in Europe and in China, coincided with the end of the local season of influenza and RSV. A report from that period already describes an early local decrement of influenza in Hong Kong, showing a 33% reduction in transmissibility based on pediatric hospitalization rate already from January 2020, when the public health measures were implemented and 98% of adults reported to wear facial masks [8].

In Europe, a study based on the national register of diseases of Finland and on the Emergency Department (ED) examinations, showed a rapid decrease in RSV and influenza cases with an early end of the epidemic season. Furthermore, in the first 4 weeks of lockdown, they reported a decrease in the number of ED visits of more than 50%, and particularly for respiratory tract infection [9].

Starting from the period of the first global spreading of COVID-19, surveillance data from the southern hemisphere showed a dramatic low level of viral respiratory infection [10], as the restrictions were implemented before the start of the local winter season. They showed a reduction, respectively, of 98% and 99.4% for RSV and influenza, despite the schools reopening. Similarly, a recent work from Brazil [11], where the winter season should be over, reports a reduction of 70% in hospitalization rate for bronchiolitis.

Regarding the winter season in the northern hemisphere, recently, a European work described the absence of RSV related bronchiolitis peak in season 2020 in Belgium, as 99% reduction in cases of RSV was reported in January 2021 [12].

As discussed by Van Brusselen et al. [12], a delayed peak of bronchiolitis could happen if the measures of individual protection and social distancing will be mitigated. If people are less exposed to infectious agents early in life, they might become more susceptible to disease later on.

Foley et al. [13] recently demonstrated an unusually delayed summer bronchiolitis and RSV peak with an older median age in Australia.

While RSV and flu seems almost absent, HRV infections remained and became prevalent, as previously reported by Wu et al. [14]. According to Takashita et al. [15], non-pharmaceutical intervention, such as environment disinfection, were responsible of enveloped respiratory virus reduction (flu and RSV), but had less effect on non-enveloped viruses circulation, such as Rhinoviruses, which are relatively resistant to ethanol containing disinfectant [16] and survive longer on surfaces [17].

Moreover, Milton et al. [18], found that surgical masks can reduce the emission of influenza virus particles into the environment by respiratory droplets, but not by aerosols. Thus, as HRV is mainly present in aerosol particles than in droplets, this could explain the minor effect of surgical masks on this virus [7].

At the time of writing (Spring 2021), Italy is still experiencing partial “lockdown” situation, with restrictions for restaurants and bars, closure of all sports and social activities, and implementation of smart working. However, nursery, kindergarten, and primary schools have been opened since September 2020, while secondary schools have been partially opened (50% of presence).

The safety of primary schools in this context is a point of extreme importance, as discussed by Villani et al. [19]. Restrictive measures, as well as the use of personal protection devices and social distancing practice by adults, had a high impact on the viruses’ epidemiology.

In this scenario, even if it is early to draw conclusions, it is possible to assume that the absence of respiratory viruses’ circulation could result in a lack of immunity against these viruses with a large cohort of immunological naïve children at major risk of contracting infection in the future.

This study presents some limitations, as it is retrospective and mono-centric. However, this report highlights an important epidemiological change due to current strategies adopted against COVID-19 and focuses on the importance to continue the active surveillance against respiratory viruses.

## 5. Conclusions

Public health measures imposed to prevent COVID-19 pandemic spread (social distancing, face masks, and non-pharmaceutical interventions) were effective at mitigating the diffusion of all respiratory infections, with a partial exception of Rhinovirus. The reduction in respiratory viruses’ circulation could result in a lack of immunity in a cohort of children and increased susceptibility to serious infections in the next seasons. Proper preparation of the health system and good education of caregivers will provide fundamental help.

## Figures and Tables

**Table 1:** Infections detected at Bambino Gesù Children’s Hospital.

	ADV	BoV	CoV	EV	Flu	MPV	PIV	RV	RSV
Season 2018–2019: total 3029									
Sep-18	12	8	7	54	0		31	128	2
Oct-18	23	15	11	43	3		43	188	8
Nov-18	42	14	23	28	0	1	55	148	26
Dec-18	64	39	59	31	10	5	52	214	248
Jan-19	39	39	56	23	105	12	28	210	287
Feb-19	32	44	53	10	122	17	20	142	155
	212	159	209	189	240	35	229	1030	726
Season 2019–2020: total 3180									
Sep-19	13	6	4	11	1	0	13	123	2
Oct-19	26	8	1	21	0	0	34	191	8
Nov-19	36	18	6	38	2	2	69	167	57
Dec-19	49	42	20	68	22	9	36	283	300
Jan-20	46	48	48	40	134	15	31	239	231
Feb-20	33	44	71	27	195	19	20	162	91
	203	166	150	205	354	45	203	1165	689
Season 2020–2021: total: 615									
Sep-20	8	3	0	8	0	0	2	63	0
Oct-20	9	7	0	5	0	0	0	135	0
Nov-20	16	3	1		0	1	4	82	2
Dec-20	20	2	0	1	0	0	2	66	0
Jan-21	10	5	2	1	0	0	2	66	2
Feb-21	10	0	2	2	0	0	0	76	1
	73	20	3	15	0	1	10	488	5

Adenoviruses (ADV), Bocavirus (BoV), Coronaviruses (CoV), Enteroviruses (EV), Influenza viruses (Flu), Human Metapneumovirus (MPV) Parainfluenza viruses (PIV), Rhinoviruses (HRV)), and Respiratory Syncytial Virus (RSV).

## Data Availability

The data used and/or analyzed during the study are available from the corresponding author on reasonable request.
